# Supplementary material for: Hemodialysis and Plasma Oxylipin Biotransformation in Peripheral Tissue
Source: Metabolites. 2022 Jan 4;12(1):34. doi: 10.3390/metabo12010034 (PMC8781597; doi:10.3390/metabo12010034)
Supplement: Supplementary file 1 [file metabolites-12-00034-s001.zip › metabolites-1534189-supplementary.pdf]

Table S1. Clinical parameters of hemodialysis (HD) patients (n=12 each)

Parameters	Patients
Glucose (60-110 mg/dl)	115.8 ± 36.7
Total cholesterol (< 200 mg/dl)	221.8 ± 181.2
LDL-cholesterol (< 130 mg/dl)	101.2 ± 30.7
HDL- cholesterol (>35 mg/dl)	42.4 ± 9.9
Triglycerides (<200 mg/dl)	151.0 ± 77.4

Data are presented as mean + SD

Table S2. Effects of hemodialysis on total plasma oxylipins in the CKD patients before (Pre-HD) and at cessation (Post-HD) of hemodialysis (n=12 each)

Amount ng/ml	pre-HD A	post-HD A	p value, t test (# paired Wilcoxon test)	pre-HD V	post-HD V	p value, t test (# paired Wilcoxon test)
	Panel A			Panel B		
**CYP epoxy-metabolites**						
5,6-EET	**25.2403 ±22.8032**	**37.0725 ±16.6846**	**0.042 #**	**16.8013 ±15.3681**	**61.8386 ±21.0905**	**0.002 #**
8,9-EET	**10.5227 ±10.8994**	**13.3085 ±5.6504**	**0.042 #**	**7.2706 ±6.6772**	**22.3969 ±7.8620**	**0.002 #**
11,12-EET	**9.9539 ±10.0802**	**14.2304 ±6.6762**	**0.036 #**	**7.0358 ±6.8420**	**25.6937 ±9.1845**	**0.002 #**
14,15-EET	**12.5615 ±12.5408**	**19.0872 ±9.2999**	**0.031 #**	**9.6655 ±9.4944**	**35.6871 ±13.5842**	**0.002 #**
5,6-DHET	1.1450 ±0.5549	1.3684 ±0.5945	0.176 #	**1.0727 ±0.5911**	**1.2782 ±0.6163**	**0.012 #**
8,9-DHET	1.7434 ±1.5788	1.6086 ±1.1218	0.902 #	**1.6063 ±1.3596**	**1.2535 ±0.9769**	**0.012 #**
11,12-DHET	0.4366 ±0.2207	0.5221 ±0.1967	0.176 #	0.4188 ±0.1959	0.4683 ±0.1888	0.056
14,15-DHET	0.4159 ±0.0996	0.4919 ±0.1251	0.124	0.3721 ±0.0788	0.4212 ±0.1045	0.07
7,8-EDP	4.5236 ±4.3719	6.0064 ±2.6897	0.097 #	**3.2930 ±3.1818**	**10.4413 ±3.5636**	**0.002 #**
10,11-EDP	4.4784 ±4.4009	6.6357 ±3.0803	0.056 #	**3.3370 ±3.0863**	**11.9332 ±3.9274**	**0.002 #**
13,14-EDP	3.3860 ±3.4990	4.8226 ±2.2061	0.097 #	**2.6621 ±2.8208**	**7.8177 ±2.6177**	**0.002 #**
16,17-EDP	2.9692 ±2.8814	3.8342 ±1.6915	0.085 #	**2.1926 ±2.0367**	**5.5352 ±2.0151**	**0.002 #**
19,20-EDP	5.5558 ±5.3859	8.6761 ±3.8647	0.056 #	**3.9685 ±3.1487**	**16.3124 ±6.2669**	**0.002 #**
7,8-DiHDPA	0.4489 ±0.2473	0.4905 ±0.2287	0.58 #	0.4110 ±0.2301	0.4461 ±0.2152	0.158 #
10,11-DiHDPA	0.1277 ±0.0387	0.1439 ±0.0452	0.371	0.1210 ±0.0346	0.1291 ±0.0539	0.475
13,14-DiHDPA	0.0892 ±0.0274	0.0989 ±0.0354	0.473	0.0846 ±0.0203	0.1040 ±0.0439	0.054
16,17-DiHDPA	0.0926 ±0.0361	0.1160 ±0.0464	0.195	0.0947 ±0.0314	0.1042 ±0.0480	0.313
19,20-DiHDPA	0.8379 ±0.4051	0.9534 ±0.4118	0.506	0.8262 ±0.3658	0.9134 ±0.4345	0.25
5,6-EEQ	0.0051 ± 0.0039	0.0084 ± 0.0052	0.069 #	**0.0034 ± 0.0026**	**0.0148 ± 0.0050**	**0.002 #**
8,9-EEQ	**1.4478 ±1.2289**	**2.6063 ±1.7732**	**0.036 #**	**0.9972 ±0.6972**	**4.3902 ±1.5836**	**<0,001**
11,12-EEQ	**0.9541 ±0.8310**	**1.8077 ±1.2340**	**0.042 #**	**0.7197 ±0.4820**	**3.4951 ±1.3735**	**0.002 #**

14,15-EEQ	0.9436 ±0.7146	1.7544 ±1.2152	0.065 #	**0.7047 ±0.4467**	**3.2728 ±1.3192**	**0.002 #**
17,18-EEQ	1.6849 ±1.3879	2.9621 ±2.1045	0.085 #	**1.3915 ±0.8490**	**6.2605 ±2.2755**	**0.002 #**
5,6-DiHETE	1.5299 ±0.9299	2.0357 ±1.4971	0.356 #	**1.3695 ±0.7134**	**1.6895 ±0.8335**	**<0,001**
8,9-DiHETE	0.0908 ±0.0378	0.1085 ±0.0552	0.389 #	0.0908 ±0.0317	0.0960 ±0.0386	0.376
11,12-DiHETE	0.0476 ±0.0379	0.0596 ±0.0600	0.255 #	0.0356 ±0.0100	0.0372 ±0.0119	0.451
14,15-DiHETE	0.0528 ±0.0201	0.0538 ±0.0249	0.916	0.0462 ±0.0135	0.0487 ±0.0203	0.458
17,18-DiHETE	0.2149 ±0.0825	0.2784 ±0.1176	0.152	0.2141 ±0.0836	0.2454 ±0.1082	0.129
9,10-EpOME	**33.0227 ±31.0831**	**51.7226 ±18.8433**	**0.023 #**	**22.5987 ±12.9602**	**93.5874 ±36.4954**	**0.002 #**
12,13-EpOME	**30.9825 ±26.7698**	**46.9109 ±15.0402**	**0.023 #**	**20.7024 ±11.0768**	**79.7224 ±23.6841**	**0.002 #**
9,10-DiHOME	4.1455 ±1.7899	5.5991 ±2.7877	0.176 #	**3.2993 ±1.7108**	**5.6467 ±2.4652**	**0.006 #**
12,13-DiHOME	4.9809 ±1.9437	6.9594 ±3.7873	0.135	**3.8968 ±1.6289**	**6.9340 ±3.3774**	**0.01 #**
**LOX metabolites**						
5-HETE	10.1945 ±2.9568	11.6499 ±3.0694	0.261	**8.9008 ±2.5521**	**11.2376 ±3.5321**	**0.01 #**
8-HETE	3.1627 ±0.8646	3.5068 ±1.3221	0.806 #	2.9701 ±0.9371	2.9656 ±1.1078	0.985
9-HETE	6.2378 ±2.4916	6.6115 ±2.5626	0.58 #	5.0812 ±2.0444	5.7522 ±2.1829	0.158 #
11-HETE	4.3689 ±1.3795	4.5454 ±1.4621	0.622 #	3.4094 ±1.1668	4.0433 ±1.4575	0.084 #
12-HETE	5.1343 ±1.7200	6.0063 ±2.3467	0.325	**5.8545 ±2.6463**	**4.7972 ±2.3597**	**0.023 #**
15-HETE	6.1984 ±1.9171	6.1869 ±1.8919	0.758 #	4.9051 ±1.5388	5.7417 ±3.2394	0.48 #
4-HDHA	2.3149 ±0.6472	2.4431 ±0.9794	0.902 #	2.1630 ±0.5977	2.5609 ±1.2943	0.388 #
7-HDHA	2.1060 ±0.6072	2.1783 ±0.8143	0.951 #	1.7319 ±0.5209	2.0061 ±0.6765	0.137
8-HDHA	1.2263 ±0.4487	1.2990 ±0.5943	0.975 #	1.0900 ±0.3821	1.1141 ±0.4935	0.875 #
10-HDHA	0.7721 ±0.2512	0.7940 ±0.3187	0.857	0.6877 ±0.2059	0.7020 ±0.2701	0.801
11-HDHA	1.0995 ±0.3769	1.0862 ±0.4291	0.758 #	1.0493 ±0.3586	1.0103 ±0.3687	0.48 #
13-HDHA	0.9168 ±0.2861	0.9169 ±0.3765	0.622 #	0.8168 ±0.2303	0.8015 ±0.3238	0.855
14-HDHA	1.1312 ±0.4384	1.1712 ±0.5441	0.849	1.0678 ±0.4203	0.9981 ±0.5062	0.158 #
16-HDHA	1.1430 ±0.3468	1.1459 ±0.4227	0.986	0.9559 ±0.3037	1.0653 ±0.4675	0.48 #
17-HDHA	1.4069 ±0.4032	1.4601 ±0.5780	0.802	1.1633 ±0.3601	1.2773 ±0.5100	0.308 #

20-HDHA	2.8698 ±0.7707	2.9700 ±0.9600	0.786	2.4726 ±0.7079	2.7939 ±1.1603	0.239 #
5-HEPE	1.4608 ±0.5576	1.5968 ±0.9534	0.902 #	1.3111 ±0.4406	1.4799 ±0.6337	0.114
8-HEPE	0.2118 ±0.0669	0.2061 ±0.1012	0.498 #	0.1959 ±0.0421	0.1817 ±0.0752	0.358
9-HEPE	0.4018 ±0.1460	0.3664 ±0.2280	0.268 #	0.3661 ±0.1209	0.3335 ±0.1318	0.182 #
11-HEPE	0.2752 ±0.0907	0.2531 ±0.1150	0.616	0.2520 ±0.0448	0.2364 ±0.0928	0.099 #
12-HEPE	0.5039 ±0.1762	0.4619 ±0.2576	0.268 #	**0.5213 ±0.1704**	**0.4038 ±0.1731**	**0.001**
15-HEPE	0.3150 ±0.1086	0.2890 ±0.1524	0.356 #	0.3142 ±0.0634	0.2596 ±0.1144	0.061
18-HEPE	0.8580 ±0.2946	0.7714 ±0.3717	0.325 #	0.8418 ±0.2239	0.7502 ±0.4476	0.117 #
9-HODE	**22.0631 ±7.3185**	**36.2855 ±21.5709**	**0.019 #**	**17.1239 ±6.0628**	**34.0942 ±21.6186**	**0.015 #**
13-HODE	**17.7783 ±6.0021**	**25.0606 ±10.8578**	**0.016 #**	**13.5081 ±4.2496**	**25.1650 ±13.2212**	**0.006 #**
CYP ω/(ω−1) metabolites						
16-HETE	0.2346 ±0.0507	0.2194 ±0.0562	0.505	0.2175 ±0.0583	0.2184 ±0.0828	0.695 #
17-HETE	0.0565 ±0.0112	0.0602 ±0.0182	0.951 #	0.0560 ±0.0145	0.0517 ±0.0147	0.198
18-HETE	0.1620 ±0.0463	0.1651 ±0.0450	0.758 #	0.1499 ±0.0282	0.1493 ±0.0421	0.952
19-HETE	0.1441 ±0.0810	0.1599 ±0.0533	0.585	0.1354 ±0.0411	0.1662 ±0.0571	0.103
20-HETE	0.4738 ±0.2011	0.4803 ±0.1680	0.934	0.3992 ±0.2211	0.4787 ±0.2014	0.138
22-HDHA	0.0994 ±0.0832	0.1067 ±0.0758	0.58 #	**0.0763 ±0.0603**	**0.1115 ±0.0824**	**0.028 #**
20-HEPE	0.1460 ±0.0808	0.1550 ±0.0963	0.81	0.1343 ±0.0692	0.1485 ±0.0768	0.413

Notes: Mean+SD. Panel A: Arterial blood. Panel B: Venous blood.

Table S3. Effects of hemodialysis on total plasma oxylipins and their ratios in venous blood of the CKD patients before (Pre-HD) and at cessation (Post-HD) of hemodialysis (n=12 each)

Amount ng/ml	pre-HD Venous	post-HD Venous	p value, t test (# paired Wilcoxon test)
5,6-EET + 5,6-DHET	**17.8740 ±15.3357**	**63.1169 ±21.0481**	**0.002 #**
8,9-EET + 8,9-DHET	**8.8769 ±6.7288**	**23.6504 ±7.8856**	**0.002 #**
11,12-EET + 11,12-DHET	**7.4546 ±6.8226**	**26.1620 ±9.1754**	**0.002 #**
14,15-EET + 14,15-DHET	**10.0376 ±9.5061**	**36.1083 ±13.6146**	**0.002 #**
7,8-EDP + 7,8-DiHDPA	**3.7040 ±3.1733**	**10.8874 ±3.5963**	**0.002 #**
10,11-EDP + 10,11-DiHDPA	**3.4580 ±3.0889**	**12.0623 ±3.9527**	**0.002 #**
13,14-EDP + 13,14-DiHDPA	**2.7467 ±2.8205**	**7.9218 ±2.6423**	**0.002 #**
16,17-EDP + 16,17-DiHDPA	**2.2873 ±2.0344**	**5.6394 ±2.0354**	**0.002 #**
19,20-EDP + 19,20-DiHDPA	**4.7947 ±3.1021**	**17.2258 ±6.4814**	**0.002 #**
5,6-EEQ + 5,6-DiHETE	**1.3729 ±0.7133**	**1.7043 ±0.8338**	**<0,001**
8,9-EEQ + 8,9-DiHETE	**1.0880 ±0.6966**	**4.4861 ±1.6000**	**<0,001**
11,12-EEQ + 11,12-DiHETE	**0.7552 ±0.4846**	**3.5323 ±1.3814**	**0.002 #**
14,15-EEQ + 14,15-DiHETE	**0.7508 ±0.4514**	**3.3214 ±1.3339**	**0.002 #**
17,18-EEQ + 17,18-DiHETE	**1.6056 ±0.8496**	**6.5059 ±2.3420**	**0.002 #**
9,10-EpOME + 9,10-DiHOME	**25.8981 ±13.6496**	**99.2342 ±38.1881**	**0.002 #**
12,13-EpOME + 12,13-DiHOME	**24.5992 ±12.1650**	**86.6564 ±25.6785**	**0.002 #**
5,6-DHET/5,6-EET	**0.0864 ±0.0565**	**0.0222 ±0.0120**	**0.002 #**
8,9-DHET/8,9-EET	**0.2858 ±0.2519**	**0.0599 ±0.0480**	**0.002 #**
11,12-DHET/11,12-EET	**0.0874 ±0.0650**	**0.0198 ±0.0099**	**0.002 #**
14,15-DHET/14,15-EET	**0.0550 ±0.0284**	**0.0127 ±0.0047**	**0.002 #**
7,8-DiHDPA/7,8-EDP	**0.5745 ±1.4719**	**0.0458 ±0.0285**	**0.002 #**
10,11-DiHDPA/10,11-EDP	**0.0514 ±0.0285**	**0.0110 ±0.0041**	**0.002 #**
13,14-DiHDPA/13,14-EDP	**0.0484 ±0.0269**	**0.0134 ±0.0039**	**0.002 #**
16,17-DiHDPA/16,17-EDP	**0.0601 ±0.0343**	**0.0194 ±0.0071**	**0.003 #**
19,20-DiHDPA/19,20-EDP	**0.2905 ±0.1983**	**0.0580 ±0.0264**	**0.002 #**
5,6-DiHETE/5,6-EEQ	**599.0058 ±431.5977**	**121.8920 ±63.0904**	**0.002 #**
8,9-DiHETE/8,9-EEQ	**0.1332 ±0.0872**	**0.0229 ±0.0107**	**0.002 #**
11,12-DiHETE/11,12-EEQ	**0.0634 ±0.0260**	**0.0112 ±0.0032**	**0.002 #**
14,15-DiHETE/14,15-EEQ	**0.0817 ±0.0318**	**0.0153 ±0.0044**	**0.002 #**
17,18-DiHETE/17,18-EEQ	**0.2159 ±0.1591**	**0.0397 ±0.0133**	**0.002 #**
9,10-DiHOME/9,10-EpOME	**0.1731 ±0.1090**	**0.0617 ±0.0222**	**0.003 #**
12,13-DiHOME/12,13-EpOME	**0.2087 ±0.0951**	**0.0877 ±0.0327**	**0.003 #**
Ratio (5,6-DHET+8,9-DHET+11,12-DHET+14,15-DHET) / (5,6-EET+8,9-EET +11,12 EET +14,15-EET)	**0.1188 ±0.0946**	**0.0253 ±0.0143**	**0.002 #**

Ratio (7,8-DiHDPA+10,11-DiHDPA +13,14-DiHDPA+16,17-DiHDPA+19,20-DiHDPA) / (7,8-EDP+10,11-EDP+13,14-EDP+16,17-EDP+19,20-EDP)	**0.1478 ±0.1106**	**0.0336 ±0.0143**	**0.002 #**
Ratio (5,6-DiHETE+8,9-DiHETE+11,12-DiHETE+14,15-DiHETE+17,18-DiHETE) / (5,6-EEQ+ 8,9-EEQ+11,12-EEQ+14,15-EEQ+17,18-EEQ)	**0.6335 ±0.4128**	**0.1315 ±0.0660**	**0.002 #**
Ratio (9,10-DiHOME+12,13-DiHOME) / (9,10-EpOME+12,13-EpOME)	**0.1904 ±0.1007**	**0.0741 ±0.0258**	**0.003 #**

Notes: Mean+SD
